# A scoping review and assessment of essential elements of shared decision-making of parent-involved interventions in child and adolescent mental health

**DOI:** 10.1007/s00787-020-01530-7

**Published:** 2020-04-16

**Authors:** Shaun Liverpool, Brent Pereira, Daniel Hayes, Miranda Wolpert, Julian Edbrooke-Childs

**Affiliations:** 1 Evidence-Based Practice Unit, Anna Freud National Centre for Children and Families, 4-8 Rodney Street, London, UK; 2 The Chicago School of Professional Psychology, Chicago, USA; 3 University College London, London, UK

## Abstract

Parents play a critical role in child and adolescent mental health care and treatment. With the increasing implementation of shared decision-making (SDM) across health settings, there is a growing need to understand the decision support interventions used to promote SDM in child and adolescent mental health services (CAMHS). The overall aim of this review is to identify and examine the existing decision support interventions available for parents. A broad search was conducted using the key concepts “shared decision-making”, “parents” and “child and adolescent mental health”. Five electronic databases were searched: PsycInfo, Embase, Medline, Web of Science and the Cochrane Library. In addition to these relevant databases, we searched the Ottawa’s Inventory of Decision Aids, Children’s Hospital of Eastern Ontario website, Google, Google Play and known CAMHS’ websites. The search identified 23 interventions available for use with parents. These interventions targeted parents providing care for children with ADHD, ASD, emotional and behavioural problems including depression (EBD), self-harm or universal mental health care. Various modalities including face-to-face, digital and paper-based versions were adopted. The majority of the interventions were able to “present options” (87%) and “discuss the pros and cons” (83%) of treatment. Time, accessibility and appropriateness of the intervention emerged as factors influencing usage and implementation of interventions. Our findings suggest that SDM interventions involving parents have been implemented differently across various presenting mental health difficulties in CAMHS. This review brings awareness of existing parent-involved interventions and has implications for the development, implementation and usage of new interventions.

## Introduction

A scoping review and assessment of essential elements of shared decision-making of parent-involved interventions in child and adolescent mental health

Mental health care and treatment decisions for children and adolescents can be challenging. In child and adolescent mental health services (CAMHS), primary caregivers (from here on referred to as parents) are confronted with many decisions. These decisions include how, when, and where to seek help [1]; agreeing on treatment options when more than one treatment option is available [2, 3]; agreeing on the goals of treatment [4, 5]; and agreeing on diagnostic tests [6]. For parents involved in the decision-making process, the journey can be complicated by a range of factors such as low levels of agreement between parents, children, and clinicians [7–18]. Decision-makers may hold different values that bring conflicting views on treatment and care options [19]. As a result, researchers and practitioners suggest that the implementation of shared decision-making (SDM) in CAMHS may be one approach to reduce treatment disagreements [20–22].

SDM is defined as the communication process that allows service users and service providers to collaborate when making care and treatment decisions [23]. SDM is considered an optimal standard to improve person-centred care and health care quality [24–26]. As such, there have been a number of initiatives to engage patients in SDM. However, in caring for children and adolescents, the decision-making process can be unique as clinicians, parents, and sometimes children are involved. Understanding the triangular relational structure [23, 27] can have implications on the implementation and development of decision support interventions.

Involving parents in decisions in CAMHS may be particularly important as many child mental health interventions require direct parent involvement. Parents may be involved as co-patients (family therapy), co-therapists [Cognitive Behaviour Therapy (CBT)], or be the direct focus of the intervention (parent training) [28, 29]. Yet to date, there has been little research on SDM involving parents in child mental health [22, 30].

A recent scoping review highlighted six approaches used in decision support interventions in CAMHS. These included: therapeutic techniques, decision aids, psychoeducational information, goal setting, discussion prompts, and mobilising patients to engage [30]. Of the 22 interventions identified in that review, 12 involved parents. However, the authors’ primary aim was not to investigate parents’ interventions in any detail but to understand what approaches existed across CAMHS. Due to the rapid increase and interest in SDM, a further review is needed to highlight specific components, such as modes of delivery and techniques that are used with various populations to promote SDM behaviour.

Gondek et al. [31] reviewed factors influencing person-centred care in CAMHS and highlighted that parental involvement positively influenced person-centred care. The authors explored published empirical studies elaborating on facilitators and barriers to person-centred care. However, SDM is a central feature of person-centred care and may present its unique influencing factors. Therefore, it is necessary to understand whether these same barriers extend to the implementation and usage of SDM interventions. Subsequent research in SDM confirms this, with young people stating that when decisions were difficult or when young people lacked capacity, parental involvement was seen as positive [85]. Hence, it was necessary to build on that review, expanding the literature search to examine the grey literature or development studies on decision support interventions.

One important step in offering decision support to parents is first to assess the decision to be made and the associated decision-making needs [32]. Providing information alone is unlikely to fully address the decision support needs of parents [33]. In attempts to promote parental involvement in child mental health decision-making, some concepts and evidence have been adopted from adult health care [34]. However, in adult settings, the decisions are usually two-way between clinician and client. In the case of a triad, the clinician, client, and caregiver/partner are usually all adults. Therefore, within CAMHS, approaches need to be tailored to accommodate varying levels of involvement depending on the child's age and capacity. Therefore, identifying appropriate decision aids would be an important step to an effective decision-making process [32].

Findings from a qualitative study indicated that the implementation of SDM in CAMHS is effortful and while tools may help support SDM, clinicians need to be allowed to use the tools flexibly [35]. Similarly, decision aids in practice have been met with various challenges [31]. Clinicians need to balance the needs of children and their parents and have complex conversations [22]. Clinicians also report being limited in their use of SDM due to service limitations, including a lack of available options, and sometimes needing to overrule decisions made by the young person due to capacity issues [86]. Therefore, examining current approaches to support parents’ involvement in SDM in CAMHS, and exploring ways to increase flexibility and usefulness of SDM, is required.

In this review, we examined the extent to which decision support interventions addressed the nine essential elements of SDM. Makoul and Clayman [36] highlighted that for SDM to occur the process should include nine essential elements: patient values/preferences, options, professional knowledge/recommendations, make or explicitly defer a decision, define/explain the problem, check/clarify understanding, explore benefits/risks, discuss patient’s ability/self‐efficacy, and arrange follow‐up. Therefore, each included intervention was assessed based on the comprehensiveness of the intervention to demonstrate these elements of SDM.

Similar reviews explored SDM from a wider perspective: interventions targeting children and clinicians, or targeting physical health [30, 31, 37, 38]. An updated review in the area of SDM in CAMHS, which focuses specifically on parent-targeted or parent-involved interventions can highlight important themes to understand parents’ involvement in the decision-making process. This is important as parents report having a better understanding of their child’s difficulties [39], and feeling better equipped to manage their child’s mental health [31] when allowed to participate in SDM.

This study aimed to conduct a systematic scoping review to identify parent-involved SDM interventions in CAMHS and assess essential elements of SDM in these interventions. A secondary objective was to explore the factors associated with implementing SDM interventions in CAMH settings.

## Research questions

The following research questions were developed to address our aims:

- What decision support interventions are available for parents of children accessing child and adolescent mental health services?
- Which of the SDM elements are addressed in these interventions?
- What are the barriers and facilitators to usage and implementation?
- What is the evidence for usefulness and acceptability of these interventions?

## Method

The methods for this review were guided by the standard review methodology [40] and those described by Arksey and O'Malley [41].

## Identifying relevant studies

The following electronic databases were searched until March 2018: PsycInfo, Embase (Ovid version), Medline (Ovid version), Web of Science and the Cochrane Library, in addition to reference lists and International Shared Decision Making (ISDM2017) conference materials. The three concepts driving the searches included “SDM”, “parents” and “CAMHS”.

In addition to the relevant databases, we searched the Ottawa decision aid list, Children’s Hospital of Eastern Ontario (CHEO) website, Google, Google Play store and known children’s mental health services’ websites. Upon completion, the empirical studies found were documented and references were imported into EndNote and all other relevant records (i.e. interventions not associated with any research literature) were added to an Excel spreadsheet.

## Selecting studies

The eligibility criteria (see Table 1) were developed alongside the research questions. Before the study began, it was agreed by SL, JEC, and MW that the elements of SDM by Makoul and Clayman [36] would be used to assess the extent to which interventions included essential elements of SDM, similar to the review by Cheng et al. [30].

**Table 1:** Inclusion/Exclusion Criteria

	Inclusion criteria	Exclusion criteria
Population	Interventions should target persons identified as being a parent/primary caregiver/legal guardian of a child with mental health problems or currently accessing child and adolescent mental health services (CAMHS)	Studies with interventions that target the parents’ illness (e.g. how a parent with breast cancer should disclose to their child who is at risk for depression) Studies/ Interventions where the parents/caregivers are not active participants in the decision-making process
Intervention	Any family/parent- targeted or parent –involved intervention tool (e.g. online decision aids, mobile applications and parent training) used by the selected population over any period of time Interventions targeted at parents/caregivers but aimed at being beneficial to decisions around the child’s mental health	The intervention is aimed only at patient medical records (e.g. databases to allow ease of access by the parents of children in CAMHs) Interventions aimed at groups with physical diagnosis (e.g. interventions for children experiencing anxieties of taking insulin) Papers where the interventions are targeted at the child and/or clinician only and excluded the caregivers
Comparator	N/A	N/A
Outcome	Intervention should aim to change levels of parental/caregiver involvement in their child’s treatment decision	Evaluating other health issues or outcomes other than mental health only (e.g. diabetes)
Study design	All study types that involve the development and testing of the intervention and published in the English Language	

Firstly, the eligibility criteria were piloted on a random sample of five papers by two independent reviewers (SL and BP). This was necessary to refine and clarify the inclusion criteria and ensure that they could be applied consistently by more than one person and reduce the possibility of rejecting relevant reports [42].

Stage 1: Once all duplications were removed, the remaining records were screened by title only and irrelevant records were excluded (i.e. records identifying physical health, e.g. asthma, or non-CAMHS settings, e.g. palliative care).

Stage 2: Abstracts were read and further records not meeting inclusion criteria were excluded. Stage 3: The remaining full-text reports and records identified through the grey literature were screened for inclusion. The most frequent reason for exclusion at this stage was the intervention not meeting any of the essential elements of SDM. All searching and screening were conducted by SL and the articles being considered for final inclusion were screened by BP to eliminate the possibility of paper selection bias. There were no major disagreements regarding inclusion/exclusion judgement and through discussion a consensus was reached to include all selected articles.

## Data extraction process

The data extraction sheet was developed based on those used in similar systematic reviews [30, 31, 37, 38]. The data were then extracted from all records being included by SL and verified by BP. Extracted variables included authors, year, target population, description of the intervention, modality, barriers and facilitators identified, study design and outcome (where applicable). Disagreements between the two investigators SL and BP regarding data extraction were resolved through discussions. Where differences in opinions for data extraction arose, a consultation was sought from JEC. A difference in opinion occurred for 3 interventions (1.3%), mainly around the identification of barriers and facilitators. We contacted two authors [43, 44] and obtained further information.

## Assessment of essential elements of SDM

The assessment of the essential elements of SDM was reported as per the number of elements of SDM characteristics met. For example, in high-SDM interventions, a higher number (7–9) of the essential elements were met, medium-SDM interventions met 4–6 of the essential elements, and low-SDM interventions met 1–3 of the essential elements. The assessments were conducted collaboratively by SL and JEC and discussed in detail before any consensus was reached. The nine elements defining SDM, according to Makoul and Clayman [36], have been used in previous studies to evaluate decision support tools [30, 87] and is one of the most frequently cited SDM models. This model was developed based on a synthesis of other SDM models and, therefore, provides a broad description of the SDM process which allows for comparisons among the identified SDM interventions [85].

## Data synthesis

The limited number of eligible RCTs and heterogeneity in the intervention type, study design, and outcomes precluded the pooling of results for a meta-analysis [45]. Therefore, a narrative synthesis approach [46] was used to address our research questions. For research questions 1 and 2, we utilised data from all the interventions identified (*n* = 23). To address research questions 3 and 4, it was only possible to include interventions that were evaluated (*n* = 15).

## Results

**Fig. 1:**
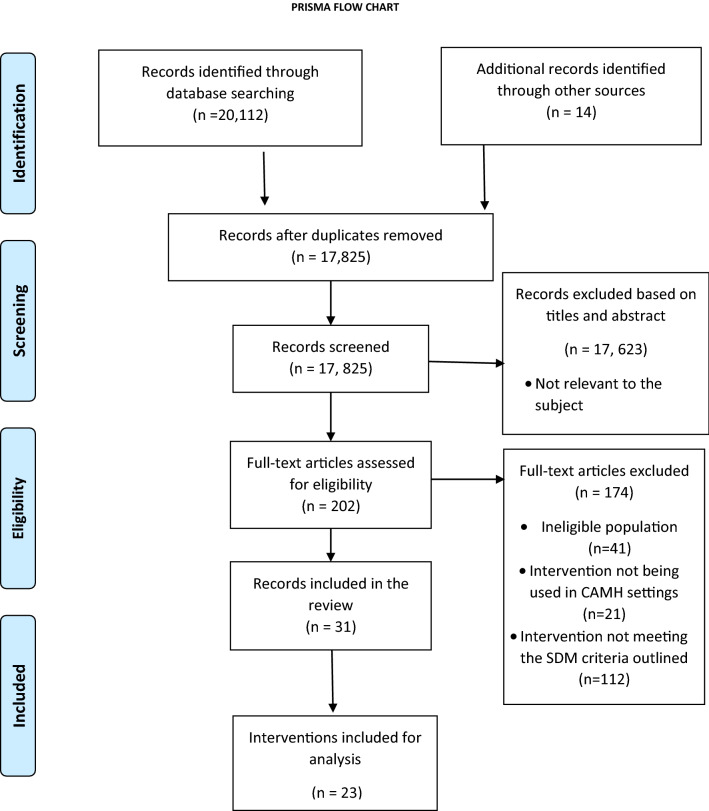
PRISMA flow diagram of study selection (adapted from Moher et al. 2009)

The database searching identified 20,112 records: PsychInfo = 3345, Embase = 7099, Medline = 5203, Web of Science = 3308 and Cochrane Library = 1157. An additional 14 records were identified through other sources in March 2018 and updated 14th December 2018: Ottawa decision aid list = 4, Reference trolling = 2, Children’s Hospital of Eastern Ontario (CHEO) = 3, Google = 5. The preferred reporting items for systematic reviews and meta-analyses (PRISMA) flow diagram (Fig. 1) depicts the flow of information through the different phases of this review and reports the number of records identified, included, and excluded.

A total of 31 records were identified for inclusion. These include 23 research articles with publication dates ranging from 1994 to 2018 and 8 interventions without any associated research publication. The interventions with development dates were developed from 2010 onwards. The 31 records identified (inclusive of development and evaluation studies), map onto 23 interventions for use by parents of children with mental health difficulties. Details related to the interventions are provided in Table 2.

**Table 2:** Characteristics of included interventions

[#]	References	Country	Intervention	Target area	*Age of child (years)	Format	Study design	Intervention description
1	Ahmed et al. [47]	Australia	Asking Questions about ADHD	ADHD	3 to 18	Paper-based	Delphi method	A question prompt list (QPL) to encourage parents to ask treatment specific questions during consultations. It contains 88 questions about the diagnosis, treatment and management of ADHD
	Ahmed et al. [79]						User-testing	
	Ahmed et al. [3]						Pre/post trial	
2	Brinkman et al. [39]	USA	ADHD SDM Intervention	ADHD	6 to 10	Paper-based	Pre/post trial	An intervention tool that includes pre-encounter cards and a booklet on ADHD treatment modalities, in addition to ADHD medication choice cards. The cards provide a brief overview of the treatment modalities including a description of the process to implement each treatment and the pros and cons of each option
	Brinkman et al. [69]						Qualitative	
3	Crickard et al. [53]	USA	The Shared Decision Framework	Universal	14 to 17	Multimodal	Preliminary user-testing	An SDM framework which includes (1) setting the stage for SDM, (e.g. training and orientation) (2) facilitating SDM (e.g. identifying decisional conflict areas) and (3) supporting SDM (e.g. process and peer support)
4	O’Brien et al. [48]	USA	Preparing for the Appointment (PFTA) worksheet	Universal	Over 10	Paper-based	Observational	This tool helps parents to identify pressing topics for discussion at the medication clinic appointment from both the parent and youth perspectives. The PFTA is designed to facilitate communication between clinician, parents and youths
5	Westermann et al. [54]	The Netherlands	Counseling in Dialogue (CD)	Universal	2 to 12	Multimodal	RCT	A semi-structured, 3-part counselling session which involves retrospection, discussing of diagnostic findings and treatment and policy arrangements. CD aims to achieve intermediate outcomes (e.g. certainty, trusts) associated with treatment success
	Westermann et al. [49]						Survey/delphi design	
6	Evans et al. [61]	USA	Families First of Essex County	Emotional and behavioural problems	Not reported	Multimodal	Quasi-experiment	A parent-driven change in the way that county services are provided to families. This service involves a range of activities, support groups & information resources to involve families in the services’ decision-making process
7	Ossebaard et al. [63]	The Netherlands	Decision Aid for ADHD	ADHD	6 to 18	Digital	Pre/Post Test	An online decision aid tapping into relevant constructs of decision making. E.g. Would you please rate your knowledge of ADHD and its treatment possibilities? This intervention contains information on different treatment options for young people with ADHD
8	Fiks et al. [80]	USA	ADHD Preference & Goal Instrument	ADHD	6–12	Paper-based	Qualitative	An instrument to assess parent’s treatment preferences and goals. This includes 3 sections addressing preferences for behaviour therapy, medication treatment and goal items
	Fiks et al. [50]						Qualitative	
9	He [56]	USA	Giving Parents a choice	Behavioural problems	4–12	Face to face	Randomized Preference Trial	An approach offering parents a choice of preferred treatment for their child. The main aim is to incorporate parents’ preferences into intervention decision-making
	He et al. [57]							
	Gewirtz et al. [55]							
10	Golnik et al. [51]	USA	ASD-specific Medical Home	ASD	0–18	Multimodal	Pre/Post Test	A service focusing on providing coordinated, comprehensive, ongoing primary care for children and young people with autism. This involved ASD care plans, change monitoring logs and tools to coordinate and improve appointments
11	Grant [44]	Australia	Interactive Early Intervention Patient Decision Aid for Parents	ASD	Under 7	Digital	Pilot RCT	A patient decision aid for parents to assist in making informed decisions about early interventions for their recently diagnosed child with ASD. The website includes general information about ASD and an interactive 8-item questionnaire that asks how important it is that an intervention improves various functional areas (e.g. academic skills)
12	Brinkman et al. [43]	USA	Coaching in deliberation	ADHD	Not reported	Face to Face	Randomized Crossover Trial	This involves an approach to strike a balance between medication benefit and side effects in addition to a decision aid that graphically depicts parent – and—teacher-reported symptoms and side effects for weekly and explicitly elicited parent preferences
13	Hayes et al. [58]	UK	i-THRIVE Grids	Low mood/ADHD/self-harm	Not reported	Paper-based	Mixed method	The grids are grounded in the THRIVE framework. It covers getting advice, getting help, and getting more help. These 8 decision aids aim to improve SDM in children and young people’s mental health
14	Barnett et al. [52]	USA	Option Grid treatment decision aid for complex behaviour problems in youth	Behavioural problems	Mean 7	Paper-based	Pilot User Testing	A one-page Option Grid patient decision aid to facilitate shared decision-making for children’s complex behavioural problems. This decision aid aims to help families and health care professionals talk about how to treat complex behaviour problems in youth ages 5 to 18
15	Carlon et al. [62]	Australia	Guided Access DVD	ASD	1–5.5	Digital	Pre/Post Test	A DVD to provide support to parents accessing and interpreting information from websites. The DVD provide guidelines for choosing interventions and provide directions on how to access websites
16	Royal College of Psychiatrists	UK	A checklist for parents with children with mental health problems	Emotional and behavioural problems	Not reported	Paper-Based	N/A	This leaflet is aimed at suggesting questions parents might ask at appointments to get information about their child’s condition
17	Autism Speaks Autism Treatment Network (n.d)	USA	Autism: Should My Child Take Medicine for Challenging Behaviour?	ASD	Not reported	Digital/Paper-Based	N/A	A Decision Aid to help parents to choose a treatment that matches the needs and values of their child and family. This tool also includes general information about ASD and prompts parents to make a decision
18	British Columbia HealthLink BC (n.d)	Canada	Depression: Should My Child Take Medicine to Treat Depression?	Depression	Not reported	Digital	N/A	A decision tool for parents/caregivers who may want to have a say in the decision. The information helps parents to understand what the choices are, so they can talk to the doctor about them
19	Healthwise Staff (n.d)	Canada	ADHD: Should My Child Take Medicine for ADHD?	ADHD	Not reported	Digital	N/A	A decision tool to provide information to help parents understand what the available choices are and to talk to the doctor about them
20	Law et al. [5]	UK	Goal progress /record / rating Charts	Universal	Not reported	Paper-based	N/A	A tool to identify and track agreed goals and monitor progress (Goal Based Outcomes). This tool allows the child/young person, parents/carer and the practitioner to discuss goals and track progress at each session
21	Agency for Health Care Research and Quality (2012)	USA	Treatment Options for ADHD in Children and Teens: A Review of Research for Parents and Caregivers	ADHD	Not reported	Digital	N/A	A summary of research for parents of a child with ADHD who may be wanting to know what the research says about ADHD. This tool addresses decision making questions
22	Agency for Health Care Research and Quality	USA	Is This Guide Right for the Child in My Care?	ASD	Not reported	Digital	N/A	A guide created to help parents talk with their child’s doctor, school administrator, social worker, or health insurance representative about available options for programs and therapies
23	Children’s Hospital of Eastern Ontario	Canada	Ottawa Family Decision Guide	Universal	Not reported	Digital/paper-based	N/A	An intervention for Families Facing Tough Health Decisions. This tool allows parents to list options, consider who is involved in the decision-making process and prompts to ask the right questions

*Age of the child is reported as the age of the children at the time the study was conducted. This do not reflect the recommended age group for use of the intervention

Question #1: What decision support interventions are available for parents of children accessing mental health services?

The 23 interventions identified in this review were: (1) Asking Questions about ADHD-Question Prompt List (QPL), (2) ADHD SDM Intervention, (3) The Shared Decision Framework, (4) Preparing for the Appointment (PFTA) worksheet, (5) Counseling in Dialogue, (6) Families First of Essex County, (7) Decision Aid for ADHD, (8) ADHD Preference and Goal Instrument, (9) Giving Parents a Choice, (10) ASD-Specific Medical Home, (11) Interactive Early Intervention Patient Decision Aid for Parents, (12) Coaching in deliberation, (13) i-THRIVE Grids, (14) Option Grid treatment decision aid, (15) Guided access DVD, (16) A checklist for parents with children with mental health problems, (17) Autism: Should my Child Take Medicine for Challenging Behaviour?, (18) Depression: Should My Child Take Medicine to treat Depression?, (19) ADHD: Should My Child Take Medicine for ADHD?, (20) Goal progress/record/rating Charts, (21) Treatment Options for ADHD in Children and Teens: A review of research for parents and caregivers, (22) Is this guide right for the child in my care?, and (23) Ottawa Family Decision Guide.

Interventions were supported by various modalities and accessible by one or more of the following formats: 43% (10) paper-based, 39% (9) digital, 17% (4) multimodal, and 9% (2) face-to-face. The majority of the interventions were available online for print, web-use, or the contact details were available to seek authors’ permission to use. The primary foci of the interventions were to support treatment decisions, highlight goals, choices and preferences, provide information, and facilitate overall doctor–client communication.

Of the 23 interventions identified, 8 were targeted at services providing care for children with ADHD, 5 were targeted at services providing care for children with ASD, 6 were for services providing care for emotional and behavioural disorders (EBD), 5 were for universal mental health care and 1 for self-harm.

Table 2 summarises the characteristics of these interventions without any hierarchical order.

Question #2: Which of the SDM elements are addressed in these interventions?

The interventions met an average of 4.57 (SD 1.93) SDM elements. Of the 23 interventions, 61% (14) included the capacity to “explain the problem”, 87% (20) to “present options”, 83% (19) to “discuss pros and cons”, 61% (14) to explore “values, goals and preferences”, 22% (5) to check service user’s “ability and self-efficacy”, 61% (14) to allow professionals to “make recommendations”, 39% (9) to “check understanding” of the available options, 39% (9) to allow users to “make or defer decision”, and 4% (1) to “arrange follow-up” if unable to make a decision at the moment or to review the decision that was made.

All of the interventions included at least two of the SDM elements. The majority (*n* = 10) of the interventions were rated as low-SDM, while 8 interventions were rated as medium-SDM, and 5 were rated as high-SDM. None of the interventions met all nine SDM criteria. Only 20% (1/5) of the interventions rated as high were evaluated, while 87.5% (7/8) of those rated as medium and 70% (7/10) of those rated as low were evaluated. The more comprehensive interventions (i.e. rated as high) included most of the elements of SDM except for “arranging follow-up”. Interventions rated as medium mostly met “explain the problem”, “make recommendation”, “present options”, “discuss pros and cons” and “explore values, goals and preferences” elements, with fewer opportunities to “discuss ability and self-efficacy”, “check understanding”, “make or defer decision” and “arrange follow-up”. Interventions rated as low mostly met “explain the problem”, “present options” and “discuss pros and cons” with some opportunities to “explore values, goals and preferences”. However, these interventions less often provided opportunities to “discuss ability and self-efficacy”, “make recommendations”, “check understanding”, “make or defer decision” and “arrange follow-up”. Table 3 summarises the results of the SDM elements checks agreed by SL and JEC.

**Table 3:** Summary of SDM elements and quality assessment

Record	Intervention	Essential elements of SDM									Assessment	
		Explain problem	Present options	Discuss pros and cons	Explore values, goals and preferences	Discuss ability and self-efficacy	Make recommendations	Check understanding	Make or defer decision	Arrange follow-up		
1	Asking Questions about ADHD	✓	✓	✓			✓	✓		✓	6	Medium
2	ADHD SDM Intervention	✓	✓	✓	✓		✓	✓	✓		7	High
3	The Shared Decision Framework	✓	✓	✓	✓			✓			5	Medium
4	Preparing for the Appointment (PFTA) worksheet	✓		✓				✓			3	Low
5	Counseling in Dialogue (CD)	✓	✓		✓		✓	✓	✓		6	Medium
6	Families First of Essex County		✓		✓		✓				3	Low
7	Decision Aid for ADHD		✓	✓			✓		✓		4	Medium
8	ADHD Preference & Goal Instrument		✓	✓	✓		✓		✓		5	Medium
9	Giving Parents a choice		✓		✓						2	Low
10	ASD-specific Medical Home	✓	✓	✓	✓	✓	✓				6	Medium
11	Interactive Early Intervention Patient Decision Aid for Parents		✓	✓	✓		✓		✓		5	Medium
12	Coaching in deliberation			✓	✓		✓				3	Low
13	i-THRIVE Grids	✓	✓	✓							3	Low
14	Option Grid treatment decision aid for complex behaviour problems in youth	✓	✓	✓							3	Low
15	Guided Access DVD		✓	✓							2	Low
16	A checklist for parents with children with mental health problems	✓	✓	✓			✓				4	Medium
17	Autism: Should My Child Take Medicine for Challenging Behaviour?	✓	✓	✓	✓	✓	✓	✓	✓		8	High
18	Depression: Should My Child Take Medicine to Treat Depression?	✓	✓	✓	✓	✓		✓	✓		7	High
19	ADHD: Should My Child Take Medicine for ADHD?	✓	✓	✓	✓	✓	✓	✓	✓		8	High
20	Goal progress /record / rating Charts				✓		✓				2	Low
21	Treatment Options for ADHD in Children and Teens: A Review of Research for Parents and Caregivers	✓	✓	✓							3	Low
22	Is This Guide Right for the Child in My Care?	✓	✓	✓							3	Low
23	Ottawa Family Decision Guide		✓	✓	✓	✓	✓	✓	✓		7	High
Total		14	20	19	14	5	14	9	9	1		

Question #3: What are the barriers and facilitators to usage and implementation?

Findings of this review suggest that factors such as time, accessibility, and appropriateness of the intervention were common themes identified as influencing usage and implementation of SDM interventions. These themes are encompassed in the two categories: facilitators and barriers.

## Facilitators

Factors influencing the usage of interventions varied across the different modalities (e.g. face-to-face vs. paper-based) and purpose (e.g. to provide information vs. to improve communication). For instance, parents expressed that they were interested in using the QPL because it was clear, easy to understand, and made it easier for them to ask questions. Most parents also indicated that the length of the QPL was “just right” and suggested that they would benefit most from the resource if it was provided soon after diagnosis [3]. Additionally, for the ADHD SDM intervention, which involved using choice cards and booklets, not having an increase in the length of the appointments was another factor encouraging usage [39]. However, feedback from families and service providers suggested that web interventions can save time, increase the efficiency of the process [53], and provide parents with information prior to sessions [54]. Parents involved in the Counseling in Dialogue study also appreciated the visualised form of information which supported their understanding. Findings across studies highlighted that knowing parents’ preferences may boost participant engagement and inform SDM [55, 56, 57].

Clinicians highlighted that one factor encouraging the use of the intervention was the minimal training requirement. Similar to parents, clinicians were also happy with no increase in the duration of consultations. Therefore, clinicians were more inclined to use the intervention if it did not affect the flow of the consultation, or strain time or staff resources [39]. Additionally, clinicians who participated in the evaluation of the i-THRIVE Grids expressed the ease of use and not detracting from practice as facilitators [58]. Another influencing factor was the clarity and appropriateness of language as indicated by participants in the study of the Option Grid treatment decision aid. That article also highlighted that clinicians appreciated interventions including information that was credible and reliable, and like other interventions, if the resources did not result in any additional time burden [59].

## Barriers

The theme of the appropriateness of the intervention was further highlighted in the article describing the Shared Decision Framework [53]. Families and service providers involved in that study expressed concerns about paperwork loads and power struggles arising from the involvement of youth in decision making [53]. Similarly, the study on the PFTA worksheets highlighted (increased) disagreement among dyads (parent and child) [60]. Findings also suggest that not giving parents a preference choice resulted in a higher chance of drop out of treatment [59].

Similar to the Shared Decision Framework, accessibility was also important to clinicians using the i-THRIVE Grids, who preferred them to be electronic for ease of access, suggesting paperwork overload as a barrier to usage [53, 58]. Another barrier to the usage of SDM interventions was highlighted in the Families First of Essex County study, which suggested that not having the availability of services and the capacity to coordinate services among their providers hindered its use [61]. Findings from the evaluation of the Interactive Early Intervention Patient Decision Aid for Parents also suggested that clinicians feared there would be a chance of information overload for parents [44]. Similar to parents’ concerns, some clinicians thought that the use of the i-THRIVE Grids and the Option Grid treatment decision aid added to the already packed schedule of service users, therefore, making them ‘burdensome’ and overwhelming [58, 59].

Question #4: What is the evidence for usefulness and acceptability of these interventions?

## Usefulness

There is evidence for 11 of the 23 interventions reporting on whether users of the interventions found it helpful or useful. Descriptions of the 11 interventions (1–5, 7, 10, 11, 13–15) are provided in Table 2. Overall, the interventions were identified as useful. Users (*n* = 17) of the QPL found it useful, and qualitative findings indicated that parents felt the QPL would address some difficulties they experienced during consultations. Parents also indicated that the booklet contained questions that were useful [3]. Early feedback from implementing the Shared Decisions Framework tools and methods indicated that youths, parents, and service providers appreciated the value in SDM and the questions on the tools [53, 60]. Similarly, the evaluation of Counseling in Dialogue resulted in parents’ understanding of the information, participation in treatment planning, and promoted an active role in decision making [54] indicating positive outcomes. Parents also described the i-THRIVE Grids as useful because the grids provided reliable information that accurately covered the range of available treatments and made them feel empowered [58].

Similar to parents, the clinicians also found the i-THRIVE grids helpful as a reminder of available options, and the users of the Option Grid treatment decision aid also indicated that the information provided was helpful [59]. More specifically, parents suggested the time in which the intervention was received was important as also suggested in relation to the Guided Access DVD, which was described as being useful for parents with a recent diagnosis [62].

The usefulness of the interventions to help parents prepare for appointments was a common theme across studies [53, 58, 59, 60, 63] as the interventions were seen as convenient, flexible, and valuable to parents’ lifestyle [53, 54]. Furthermore, the evaluation of the Counseling in Dialogue intervention found that the visualization elements of the intervention were helpful in supporting parents’ understanding of the information [54] and the Interactive Early Intervention Patient Decision Aid for Parents pilot study highlighted that some parents found the intervention overall useful [44], The usefulness of the intervention was further highlighted by parents in the evaluation of the ASD-specific Medical Home intervention who reported experiencing fewer unmet needs, and an improvement in SDM (5.89 vs 4.03, *p* < 0.05) than the control group. However, that study reported marginal statistical significance between the groups for unmet needs (5.95 vs 5.17, *p* = 0.067) [64].

Clinicians indicated that the QPL helped parents initiate discussions about difficult topics and helped (or will help) parents in making decisions [3]. Overall, 71% of physicians in the evaluation of the ADHD SDM choice cards and booklets found the information extremely helpful and acceptable for use by parents [39]. Similar to parents, the therapist also considered the Counseling in Dialogue intervention to be a convenient and valuable method [54] and clinicians in the qualitative study of the i-THRIVE Grids suggested the grids were useful in the context of assessment clinics and ‘intrinsically useful’ to service users [58]. Clinicians also found the Option Grid treatment decision aid useful in structuring the session and reducing the burden related to paper handouts [59].

## Acceptability

Eight of the twenty-three evaluated interventions reported on acceptability. Descriptions of the 8 interventions (1, 2, 4, 7, 10, 13–15) are provided in Table 2. The interventions were generally acceptable by users. For example, the QPL was well-received by participants in the study and resulted in a mean satisfactory score of 9.5 on a 10 point scale measure. Results showed that all parents were very satisfied or satisfied with the use of the QPL. The paediatricians also agreed that the QPL was acceptable for use by families and indicated that they would be happy to use it as part of their practice [3]. In the evaluation of the choice cards and booklets of the ADHD SDM Intervention, physicians indicated the resources were acceptable for use by families and 86% indicated that they would recommend it [39]. Similarly, parents responded positively to using the PFTA worksheets and despite some parents reporting moderate levels of satisfaction, some were eager to use it again for future appointments [13].

The decision aid for ADHD received average feedback ratings on whether users were satisfied with the decision aid itself and users reported moderate satisfaction with the information received via the tool [63]. Participants in the intervention group for the ASD-specific Medical Home study were more satisfied than those in the control group (6.49 vs 4.98, *p* < 0.01) [64]. Additionally, a parent in the qualitative study of the i-THRIVE Grids highlighted satisfaction with the intervention as it ‘allowed her to make the decision that was right for her family’ [58]. All participants using the Guided Access DVD indicated that they would recommend the intervention to others and some of the parents highlighted that they were very likely to continue using the tool [62]. Although interventions were acceptable, some parents and clinicians who used the Option Grid highlighted that the resources needed to be used during sessions because as a stand-alone intervention parents may feel overwhelmed by the amount of information [59].

## Discussion

This scoping review was designed and carried out to identify and examine parent-targeted SDM interventions to inform practice and the development and implementation of future decision support tools. This study identified a total of 23 interventions for use by parents of children with mental health difficulties. The findings of this review suggest that interventions targeting parents met on average 4.57 (SD 1.93) essential elements of SDM and have received favourable responses to usage (acceptability and usefulness). The factors influencing usage and implementation of the interventions emerged as three overarching themes: time (e.g. increase in session times), accessibility (e.g. easily available via the web), and appropriateness of the intervention (e.g. easy to use and understand).

The review by Cheng et al. [30], examining SDM interventions for children and young people, also identified 12 of the interventions that our study found, and conducted similar quality checks using the Makoul and Clayman [36] elements which coincide with our findings. However, it must be noted that the nine elements of SDM were developed based on the literature reviewed in adult physical health settings. Therefore, applying this model to CAMHS may require more involvement from service users within CAMHS to understand how to include these elements in the interventions. With the uniqueness of the triad in CAMHS, even more research is needed to ensure these elements can be included in the development of interventions to support the SDM process. Additionally, the higher number of interventions “presenting options” but fewer “arranging follow up” can be explained as an immediate approach to medicinal decision making, which is mostly required in physical health. With more chronic conditions in mental health, the “arrange follow up” component may be quite useful for this population and developers can consider this going forward.

Interventions were targeted at services providing care for children with ADHD, ASD, EBD, universal CAMHS, or self-harm. This finding is also consistent with previous reviews [30, 65], highlighting that most interventions in CAMHS target these disorders. This is not surprising as statistics show one in eight (12.8%) of 5–19-year-olds have at least one mental health disorder [66]. Additionally, findings from a 30,000 children study found that just under one in five children and young people indicated they were experiencing emotional problems and the same was seen for behavioural problems [67]. Therefore, it is noted that parents of children with these mental health difficulties will be faced with making a wide range of decisions.

Previous research in this area highlighted barriers and facilitators to person-centred care in CAMHS [31]. However, this review aimed to investigate further, to discover if there were any factors specific to the use of SDM interventions by parents. Findings were consistent with the previous literature in both physical and mental health regarding the general importance of information sharing [31, 68] as a facilitator. Parents appreciated having information from a variety of sources to help make decisions [33, 39]. However, as this review also highlighted, the information should be appropriate, for example in a language that is jargon-free and understandable for service users [31, 68]. Knowing the types of information parents need and how to use the right media to effectively communicate the relevant information can aid parents in decision making [69, 70, 71].

Another facilitator highlighted was time efficiency, for example in being able to prepare for appointments ahead of the session. This can be favourable to parents as they are usually faced with long waiting times and time-consuming evaluations [72]. Therefore, the time spent waiting will be occupied with preparations for upcoming appointments. Additionally, accessibility of the interventions was important, for example, some parents found web-based interventions to be appealing. Although there is growing evidence to support technology in health care settings [73], more evidence is needed to investigate parents’ preference for using digital interventions as a stand-alone or integrated into face-to-face sessions to support their children. From the clinician's perspectives, SDM support interventions were likely to be used if they required minimal training and had no increase in the duration of the consultations. There has been little research to date concerning clinicians’ time as a resource [74]. However, there have been increasing emphasis on time and efficiency in health care delivery [75]. Therefore, having interventions that can be used during and within sessions can impact both clinicians’ and parents’ satisfaction with services by increasing efficiency.

In line with previous findings from similar reviews, not all interventions identified were evaluated [30, 38]. This study found that 15 (65%) of the included interventions had associated research publications. Therefore, reporting on usefulness and acceptability for all interventions is limited, and it is, therefore, difficult to recommend their use. The increase in commercially developed interventions leaves empirical studies lagging behind. This is concerning, given the emotional state of this population. Parents of children with mental health issues report high levels of parenting stress [76] and, therefore, caution should be taken when implementing new interventions to ensure sufficient support is given throughout the decision-making process. Rigorous and ecologically valid empirical studies should be conducted to test these interventions before implementing into practice.

Service users and service providers found interventions to be useful for the decision-making process. This is consistent with existing literature as SDM has been widely advocated across health settings, patient populations and policy [22, 24]. One reason highlighted for the usefulness of the interventions was the ability to provide or facilitate information sharing. This again corroborates previous findings that information seeking is a primary element of the journey parents undergo post-diagnosis of a child with a mental health disorder [77]. However, it is noted that information needs may change at different periods [44] and information only may not be sufficient for parents [33]. Therefore, additional support needs should be offered at various stages.

Similarly, clinicians found interventions to be useful as it facilitated discussion. In pediatric health settings, health professionals welcome additional resources that provide access to information at the convenience of parents, and outside of the clinical session [78]. As a result of this, parents can be better prepared for appointments allowing for further discussions between parents and clinicians. In CAMH settings, similar findings indicate that keeping reports and tracking progress leads to shared work between the therapist, young person and family, which can lead to better agreement and working alliance in therapy [5].

Eight interventions had supporting evidence to indicate overall satisfaction with the use of the intervention. This is supportive of previous studies that highlight parents’ need for additional support [79, 80] to make informed decisions. Therefore, the findings of this review confirm that parents were satisfied with receiving more information through SDM interventions. These findings suggest that once parents are provided with the right kind of support, they will feel more included by services and their own anxieties of not being informed will decrease [81]. Clinicians also responded favourably to using SDM interventions suggesting that services have a willingness to implement PCC as recommended in policy guidelines for health care [24, 25, 82].

## Strengths and limitations

This review has major strengths, such as, including a very broad search strategy similar to those already published [30, 31] and a comprehensive concept-specific tool for assessing essential elements of SDM [36]. However, there are some limitations to be considered when interpreting the findings of this scoping review. Firstly, of the 23 interventions, only 9 were identified through the database searching. This can be due to the lack of a standardized definition (e.g. decision aid, decision support tools, and decision support interventions) used for SDM [36]. Although this review used a very broad search strategy and two independent reviewers, it was possible that some records may have been missed. Secondly, not all the interventions identified were evaluated and those that were evaluated lacked homogeneity in terms of study design, SDM outcome measure, mode of delivery, and target population making it difficult to synthesize.

For this review, we examined the essential elements of SDM in interventions using the framework by Makoul and Clayman [36]. Although these guidelines are useful in providing an overall sense of whether the intervention is achieving its purpose, the behaviours associated with each criterion may differ making it difficult to standardize [83]. Additionally, the lack of detail and heterogeneous study designs made it difficult to objectively conduct an assessment using this tool as it was uncertain how the intervention was used within the client–clinician interactions. An alternative assessment tool that can be considered in future studies is the International Patient Decision Aid Standards (IPDAS), which provides a minimal set of standards for qualifying as a decision aid, and for judging the quality of decision aids [84]. However, the IPDAS may not have been suitable for the current study as the authors aimed to assess the presence of essential elements of SDM in relations to the SDM process and not the quality of the intervention itself. Assessing the quality of the evidence underlying the interventions, including development and evaluation, may have required contacting the interventions’ developers, which was beyond the scope of this review. Furthermore, the results of this review are only up to date as of December 2018. Nonetheless, it is believed that this scoping review provides important information, and it is the most rigorous in the area of parent-targeted SDM in CAMH settings that the authors are currently aware of.

## Future directions and recommendations

There is an urgent need for adequately powered and rigorously designed RCTs to evaluate the efficacy of parent-targeted SDM support interventions. Conducting such studies can support researchers in identifying and comparing specific elements that best support the SDM process in future review studies. Based on findings from this review, some broad key recommendations are suggested to develop and implement SDM support interventions. First, it is recommended that interventions not reaching IPDAS criteria [84] report on elements of SDM involved in the intervention, so end users can obtain additional support to supplement the intervention if needed. Second, as identified by some service users and service providers, interventions should be web-based or online to avoid paperwork overload. Just as important, it is recommended that new interventions require minimal training for both providers and users of the interventions and that the interventions be made accessible via an open access repository of SDM interventions. Another recommendation is that the content and usage of the interventions be easy to understand. Finally, it is recommended that service providers receive the necessary support and knowledge to be confident in recommending or using decision support tools with service users.

## Conclusion

In conclusion, this scoping review provided a broad overview of parent-targeted decision support interventions used in CAMHS. It is noted that further research is needed to evaluate and compare parents’ preferences for decision support interventions. At a minimum, this review may serve to provide awareness of available parent-involved SDM support interventions and inform guidelines for the development, implementation, and usage of new interventions.

## Abbreviations

ADHD: Attention deficit hyperactivity disorder
ASD: Autism spectrum disorder
CAMH: Children and adolescents mental health
CAMHS: Child and adolescent mental health services
CBT: Cognitive behavioural therapy
EBD: Emotional and behavioural problems including depression
IPDAS: International Patient Decision Aid Standards
NHS: National Health Service
PCC: Person-centred care
PFTA: Preparing for the appointment
QPL: Question prompt list
RCT: Randomised controlled trial
SDM: Shared decision-making
